# Solvent-surface interactions control the phase structure in laser-generated iron-gold core-shell nanoparticles

**DOI:** 10.1038/srep23352

**Published:** 2016-03-23

**Authors:** Philipp Wagener, Jurij Jakobi, Christoph Rehbock, Venkata Sai Kiran Chakravadhanula, Claas Thede, Ulf Wiedwald, Mathias Bartsch, Lorenz Kienle, Stephan Barcikowski

**Affiliations:** 1 Technical Chemistry I and Center for Nanointegration Duisburg-Essen (CENIDE), University of Duisburg-Essen, Universitaetsstrasse 7, 45141 Essen, Germany; 2 AG-Synthesis and Real Structure, Institute for Materials Science, Faculty of Engineering, Kiel University, Kaiserstrasse 2, 24143 Kiel, Germany; 3 AG-Inorganic Functional Materials, Institute for Materials Science, Faculty of Engineering, Kiel University, Kaiserstrasse 2, 24143 Kiel, Germany; 4 Faculty of Physics and Center for Nanointegration Duisburg-Essen (CENIDE), University of Duisburg-Essen, Lotharstrasse 1, 47057 Duisburg, Germany

## Abstract

This work highlights a strategy for the one-step synthesis of FeAu nanoparticles by the pulsed laser ablation of alloy targets in the presence of different solvents. This method allows particle generation without the use of additional chemicals; hence, solvent-metal interactions could be studied without cross effects from organic surface ligands. A detailed analysis of generated particles via transmission electron microscopy in combination with EDX elemental mapping could conclusively verify that the nature of the used solvent governs the internal phase structure of the formed nanoparticles. In the presence of acetone or methyl methacrylate, a gold shell covering a non-oxidized iron core was formed, whereas in aqueous media, an Au core with an Fe_3_O_4_ shell was generated. This core-shell morphology was the predominant species found in >90% of the examined nanoparticles. These findings indicate that fundamental chemical interactions between the nanoparticle surface and the solvent significantly contribute to phase segregation and elemental distribution in FeAu nanoparticles. A consecutive analysis of resulting Fe@Au core-shell nanoparticles revealed outstanding oxidation resistance and fair magnetic and optical properties. In particular, the combination of these features with high stability magnetism and plasmonics may create new opportunities for this hybrid material in imaging applications.

One focus in nanotechnology is to combine nanoparticle properties, such as nanomagnetism, plasmonics, and thiol-based conjugation chemistry, to generate multifunctional nanomaterials^1^. This can be achieved by combining two or more metal components into a single alloy or core-shell nanoparticle^2^,^3^. For biomedical applications, the gold-iron system is particularly interesting^4^,^5^,^6^,^7^. In this context, iron-based particles are relevant because of their magnetic properties that are applicable for magnetic resonance imaging (MRI)^8^ and thermotherapy^9^, whereas gold is a promising material for its oxidation resistance, optical properties^10^, and ability to be easily functionalized by thiolated biomolecules^11^. Furthermore, the concept of dual MRI/optical imaging has been discussed in the literature^12^,^13^. However, the combination of gold and iron within a single nanoparticle is non-trivial, as the phase diagrams of gold and iron are complicated and include a broad miscibility gap^14^. In addition, non-noble iron also suffers from oxidation, making the controlled formation of FeAu alloy nanoparticles even more challenging. Next to particle composition, the internal phase structure of alloy nanoparticles is also a critical determinant controlling particle properties. In the case of medical applications, a core-shell structure^15^,^16^,^17^,^18^,^19^, comprising an inert noble metal shell (such as gold) protecting a reactive magnetic core (such as iron) from oxidation, is the most promising strategy^20^. Conventional syntheses of binary nanoparticles are based on the co-precipitation of metal salts^21^ or reverse micelle methods^13^,^22^. In these cases, the chemical synthesis of Fe@Au core-shell nanoparticles can be achieved; however, inchoate oxygen-permeable Au shells formed, which led to slow oxidation of the Fe cores within several days^22^. However, chemical synthesis routes are frequently complicated by numerous factors: I) a lack of suitable educts for specific synthesis approaches, II) impurities from precursors^15^, and III) incomplete conversion into uniform particles because of different reactivities and chemical equilibria^22^,^23^. More detailed information concerning the chemical synthesis of multi-material nanoparticles can be found in a recent review by Cortie and McDonagh^24^.

Aside from chemical methods, several studies have also reported on the utilization of physical methods for FeAu nanoparticle synthesis. Amram *et al*. showed the synthesis of Fe@Au core-shell nanoparticles by the solid-state dewetting of thin Fe-Au bilayer films deposited on a sapphire substrate^25^, and Velasco *et al*. prepared FeAu alloy nanoparticles by inert-gas condensation for biomedical applications^26^. Among the physical synthesis routes, pulsed laser ablation in liquids (PLAL) is one of the most promising methods for nanoparticle synthesis, as it produces pure and uniform alloy nanoparticles and may efficiently overcome some of the problems arising during chemical synthesis^27^. In this context, PLAL is a well-established method, and colloidal nanoparticles from numerous binary alloy targets, including AgAu, FeNi, NiTi, PtIr, and FeAu, in different liquids have been fabricated by this technique^27^,^28^,^29^,^30^,^31^,^32^,^33^,^34^. This method allows the generation of surfactant-free nanoparticle colloids without the use of chemical precursors or toxic preservatives, which could be beneficial for potential biomedical applications. The formation of solid-solution alloy nanoparticles by laser synthesis has been previously reported^27^,^31^, including detailed studies of particle morphology in the case of monophasic solid-solution AgAu particles^32^. A gold-iron nanoparticle system made by laser ablation in solution was intensively studied by Amendola *et al*.^28^,^29^,^30^,^35^. They showed how to combine the optical properties of gold, such as surface-enhanced Raman scattering (SERS), with magneto-responsive iron and iron oxide in ligand-free nanoparticles. An example of these materials’ potential for biomedical applications was given by the magnetic sorting of murine macrophages with enclosed AuFeO_x_-nanoparticles and the subsequent SERS-imaging of the sorted cells^36^. In another approach, the impact of the surrounding medium on the morphology of laser-generated FeAu nanoparticles was studied. Here, in ethanol, non-segregated plasmonic alloy nanoparticles were obtained; however, oxide formation was reported after the addition of 0.2% of H_2_O or H_2_O_2_ to ethanol^37^.

Although the formation of FeAu alloy particles via PLAL has been previously studied, a detailed understanding of the formation mechanism and a clear correlation between the composition of the surrounding medium and particle internal phase structure remain lacking. To fill these gaps, this study systematically examines the PLAL of FeAu targets in the presence of aqueous and organic media. Detailed high-resolution transmission electron microscopy (HR-TEM) and energy-dispersive X-ray spectroscopy (EDX) analyses are utilized to probe the internal structure and composition of the nanoparticles.

## Methods

The laser-generated nanoparticles were obtained by using a femtosecond laser (Spitfire Pro, Spectra-Physics) with a central wavelength of 800 nm, a pulse duration of 120 fs, a pulse energy of 300 μJ, a repetition rate of 5 kHz, and a focal distance of 150 mm. The target (Fe_44_Au_56_ alloy, custom made by the “Research Institute for Noble Metals and Metal Chemistry”, Schwaebisch Gmuend, Germany) was placed at the bottom of a vessel filled with 4 mL of solvent (i.e., deionized water, technical-grade acetone with 99.5% purity, or methyl methacrylate (MMA), purchased from Sigma-Aldrich with 99% purity). The liquid layer above the target was 5 mm thick. A 5-axis manipulator moved the vessel with a speed of 1 mm/s in a spiral pattern for a total laser ablation time of 10 min. The influence of the pulse duration was investigated using a picosecond pulsed laser (Atlantic 532, Ekspla) with a wavelength of 1064 nm, a pulse duration of 10 ps, a pulse energy of 160 μJ, a repetition rate of 100 kHz, and a focal distance of 100 mm, and a nanosecond pulsed laser (PowerLine 20 E, Rofin) with a wavelength of 1064 nm, a pulse duration of 8 ns, a pulse energy of 0.8 mJ, a repetition rate of 15 kHz, and a focal distance of 100 mm. The hydrodynamic size and zeta potential of the resulting colloidal solutions were characterized by dynamic light scattering (DLS) using a Zetasizer (ZS, Malvern). High-resolution transmission electron microscopy (HR-TEM) was performed using a Tecnai F30 G^2^ ST instrument equipped for EDX analysis. Further elemental mapping using energy-filtered TEM with a Gatan image filter Tridiem was also performed to confirm the results. The functionality of the nanoparticles in terms of their optical and magnetic properties was analyzed. The optical properties were studied within the wavelength range of 350–800 nm using ultraviolet/visible (UV/Vis) absorption spectroscopy (Shimadzu 1650) using a quartz cuvette (Helma Analytics) with a path length of 10 mm. The magnetic properties of the nanoparticles produced by ablation in acetone were studied using a vibrating sample magnetometer (VSM, Lakeshore Model 7300). A sample for VSM measurements was prepared by drying 100 μL of a nanoparticle dispersion (mass concentration = 374 μg/mL) on pieces of filter paper that were 5 mm × 5 mm in size. Focused ion beam (FIB) techniques (FEI Helios Nanolab combined Focused Ion Beam/SEM) were used for the preparation of a thin lamella containing solid nanoparticles. The FIB-milled nanoparticles were analyzed using TEM-EDX techniques and an FEI Tecnai F20 instrument. Details concerning the FIB techniques and the milling procedure of the solid nanoparticles are presented in the Supplementary Information. The nanoparticles’ chemical stability was probed using chemical etching protocols involving hydrochloric acid. First, the colloidal nanoparticles were exposed to 10% HCl for 1 h and subsequently magnetically separated and washed with deionized water. In the second step, etching was conducted with concentrated HCl (37%, for 1 h), and the samples were washed three times and magnetically separated.

## Results and Discussion

**Figure 1:**
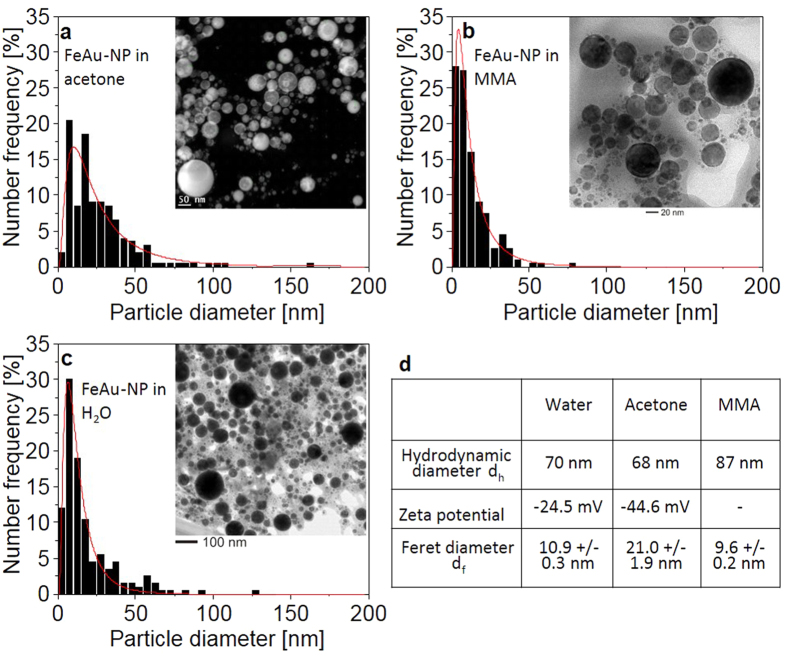
Size distributions of FeAu alloy nanoparticles generated in (**a**) acetone, (**b**) MMA, and (**c**) water. The number-weighted particle size distributions were fitted with a log-normal function. (**d**) Hydrodynamic diameters (dh), zeta potentials (ξ), and Feret diameters (df) of FeAu nanoparticle colloids in different solvents (ξ could not be measured in MMA because of high viscosity). The df values were obtained from the number mean values of the log-normal fitting functions.

The laser ablation of FeAu targets was conducted in three different solvents—water, acetone, and MMA—and in all cases, stable nanoparticle colloids were obtained. The stability was probed using time-resolved UV-Vis spectroscopy measurements. The spectra clearly reveal that the extinction at λ = 520 nm of the colloids formed in acetone decreased by <8% over a period of 21 days, indicating good colloidal stability. Furthermore, the extinction recorded in the near-infrared (NIR) range (λ = 800 nm) was completely unchanged, verifying that no additional agglomerates, which are prone to show intensive scattering in this spectral regime, formed in the respective time period (Fig. S13 in the Supplementary Information). Subsequently, the generated colloids were characterized using DLS and TEM. The resulting Feret diameters, hydrodynamic diameters, and zeta potentials are shown in Fig. 1d. Furthermore, the particle size distributions obtained from the TEM images are depicted in Fig. 1a–c. Although the hydrodynamic diameters (d_h_) of the colloids generated in all three solvents were very similar, the Feret diameters (d_f_) significantly deviated. In all cases, d_h_ was larger than d_f_, possibly because particle analysis techniques, such as DLS, overestimate larger particles and small agglomerates in solution and cannot properly characterize small particle fractions in the presence of large ones. In addition, the TEM images (Fig. 1) seem to indicate the presence of agglomerated primary particles. Based on these data, whether these agglomeration processes are artefacts of drying on the TEM grids or whether agglomerates are present in solution remains unclear. However, a contribution of agglomerates formed during the ablation process to the hydrodynamic diameter measured via DLS cannot be excluded. These agglomerates would be detected as individual particles by DLS and may explain why d_h_ was larger than d_f_.

Furthermore, the particle sizes (d_f_) of FeAu in acetone were significantly larger than those found in MMA and water. Particle size distributions during PLAL are dominated by particle growth-quenching mechanisms, inducing the stabilization of reactive particle surfaces by solvent molecules. In aqueous solutions, this phenomenon is governed by anion adsorption^38^,^39^ in the case of noble metals, such as Au. However, in non-noble metals, such as iron, surface oxidation and the formation of iron hydroxide shells have been reported to be key factors driving particle stabilization^37^. In the presence of organic solvents, the formation of enolates and alcoholates, which may polymerize on the particle surface, can induce stabilizing effects^40^,^41^. A detailed discussion of this phenomenon in the context of particle phase structure can be found below.

The zeta potentials of the FeAu nanoparticles synthesized in acetone and an aqueous solution deviated significantly, with the acetone-based solution exhibiting more negative values. Although highly negative zeta potential values are typical for metal colloids obtained by PLAL^27^,^42^,^43^, the pronounced differences in zeta potentials between the colloids obtained in water and acetone indicate deviating surface charge densities. This may be evidence of pronounced differences in the particle surface chemistry caused by different surface compositions. Because further interpretation of these data would require a detailed knowledge of the particle’s elemental composition, the internal phase structure of the nanoparticles was further characterized using high-angle annular dark field scanning transmission electron microscopy (HAADF-STEM) in combination with EDX.

**Figure 2:**
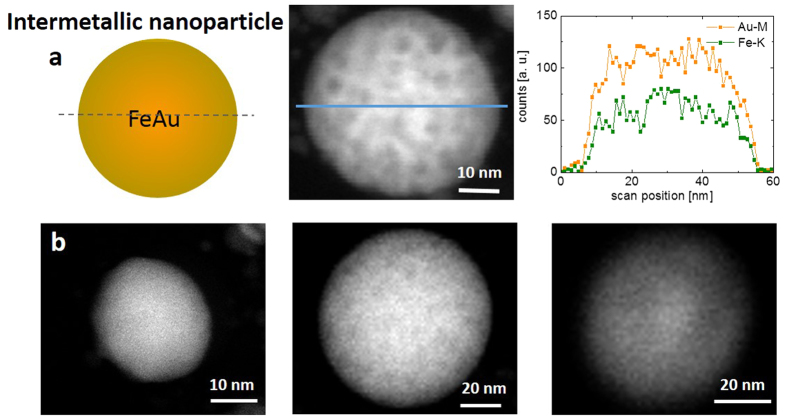
(**a**) SETM-HAADF EDX line scan of an intermetallic (solid-solution) nanoparticle. (**b**) STEM-HAADF images of intermetallic nanoparticles that were laser-generated in acetone.

**Figure 3:**
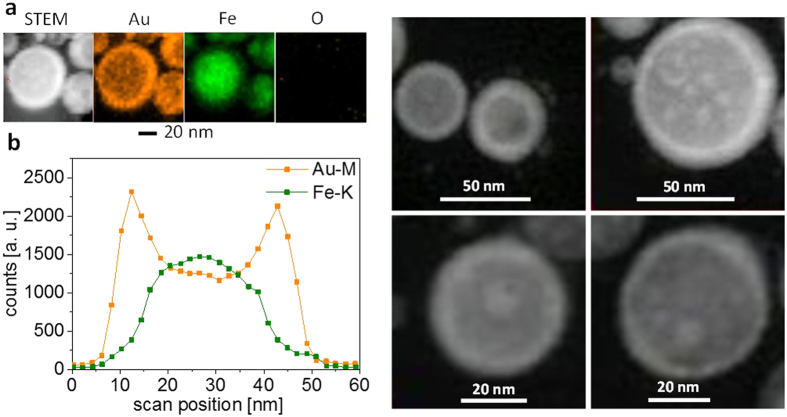
(**a**) STEM-HAADF images providing z-contrast (left and right images) and STEM-EDX elemental maps of nanoparticles from the laser ablation of FeAu in acetone (for MMA, see Supplementary Fig. S2), confirming the presence of the non-oxidized iron core and gold shell. Line scans (**b**) of the nanoparticle in (**a**) showing the core-shell nature.

**Figure 4:**
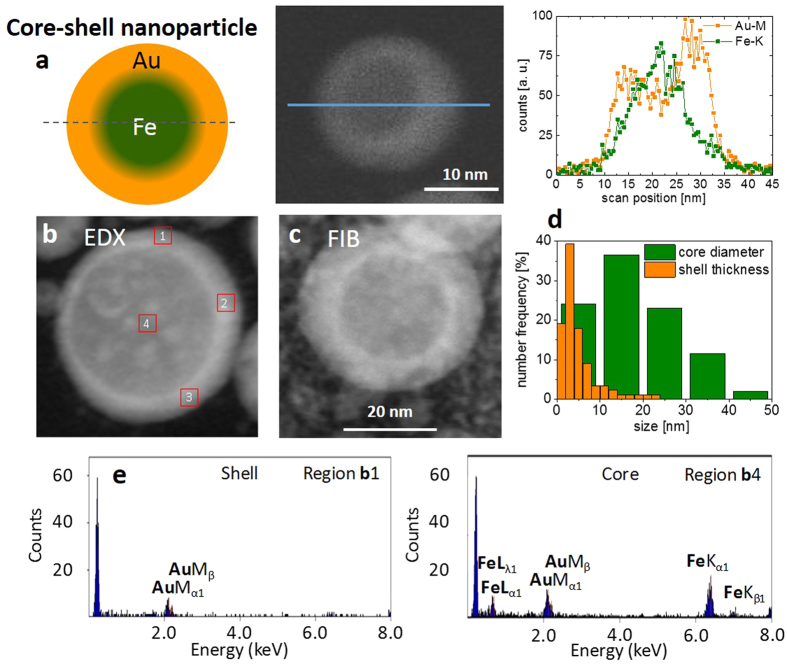
Analysis of core-shell nanoparticles that were laser-generated in acetone. (**a**) Line scan and EDX signal of a core-shell nanoparticle. (**b**) STEM-HAADF image of an Fe@Au core-shell nanoparticle with regions of EDX analysis indicated (shell: regions 1–3, predominantly Au, core: region 4, composed of Fe and Au. For the detailed EDX analysis, see Supplementary Fig. S6). (**c**) STEM–HAADF image of an FIB-milled Fe@Au core-shell nanoparticle. (**d**) Histograms of the shell thickness and core diameter of laser-generated Fe@Au core-shell nanoparticles. Average values and peak maxima indicate a mean core diameter of 15 nm and a shell thickness of 3 nm. (**e**) Representative EDX point measurements indicating the presence of pure Au in the shell (region b1) and a mixture of Au and iron in the core (region b4).

The analysis of the morphology revealed interesting properties. In all solvents, bimetallic FeAu nanoparticles were generated, which showed a pronounced core-shell structure. Intermetallic nanoparticles with homogeneous elemental distributions formed less frequently (Fig. 2) and were only found in <10% of all examined particles. However, an elemental analysis of FeAu nanoparticles by EDX on the single-particle level revealed an atomic composition of Fe_47_Au_53_ (average of five point measurements), which was similar to that of the target material (Fe_44_Au_56_, Supplementary Table S1 and Fig. S9). Therefore, although the overall composition of the particles remained similar in all experiments and was close to the composition of the corresponding target (Fe_44_Au_56_), the structure and morphology of the core-shell material clearly differed depending on the solvents applied during laser ablation. In the case of PLAL in acetone and MMA, a gold shell formed around a non-oxidized iron core (Fig. 3a,b, and Supplementary Fig. S2). In contrast to chemically derived Fe@Au nanoparticles^22^, which were subject to rapid oxidation of the iron core, in the present study, EDX revealed no oxygen in the metal core, even after several weeks of storage. Thus, the Au shell of the laser-generated nanoparticles protects the reactive Fe core against oxidation. These findings were clearly confirmed by the EDX analysis (Fig. 3b). The majority of the binary nanoparticles exhibited a mean shell thickness of 3 nm and a core diameter of 15 nm (Fig. 4d). These findings were further verified by line scans (Fig. 4a and Supplementary Fig. S8). The differences between the core-shell and homogeneous alloy nanoparticles could be further clarified by normalizing the element-specific signal to the total signal (Supplementary Figs. S4 and S5). These results conclusively demonstrate that the majority of the examined particles formed a defined core-shell nanostructure with iron enrichment in the core and gold enrichment in the shell without any traces of oxygen. However, it should be noted that the elemental segregation might not be absolute. Additional EDX analysis of different nanoparticle regions (Fig. 4b) clearly revealed that no significant quantities of iron were present in the shell (Fig. 4e, Supplementary Fig. S6, regions 1–3); however, naturally, both iron and gold were found when the whole particle, shell and core, (region 4) were analyzed. Based on these measurements, we cannot exclude the presence of low amounts of gold in the core, but the formation of an iron-rich core material was clearly indicated. These findings are in good agreement with recent literature, where an Fe-Au core-shell structure synthesized by chemical methods was reported based on similar EDX measurements^44^,^45^. To validate this unique structure of a crystalline gold shell protecting an elemental iron core, we measured the EDX line scans of a focused ion beam-prepared cross-section through some nanoparticles (Supplementary Figs. S7 and S8). This technique confirmed the presence of a characteristic elemental distribution of a core-shell phase structure after FIB cutting, with a sharp intra-particle interface between the shell and the core imaged, as shown in Fig. 4c. However, it should be noted that, based on the line scans, the presence of low amounts of iron in the shell and low amounts of gold in the core cannot be excluded, although a significant elemental enrichment is obvious. In a recent publication, Scaramuzza *et al*. applied X-ray photoelectron spectroscopy and showed that an FeAu shell containing oxidized iron atoms also contributed to the stabilization of an iron-rich core^37^. As the EDX measurements conducted in this study were not surface sensitive, the formation of an FeAu instead of a pure Au shell cannot be excluded.

**Figure 5:**
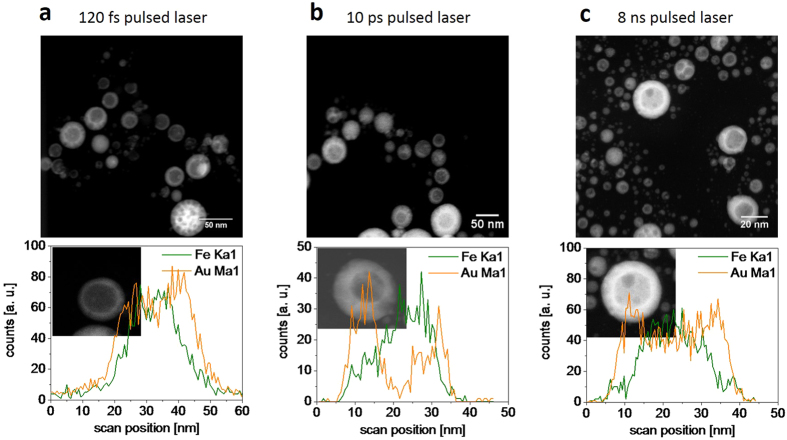
HAADF-STEM images and EDX line scans of Fe@Au core-shell nanoparticles generated by ablation with different laser pulse durations in acetone. (**a**) 120 fs, (**b**) 10 ps, and (**c**) 8 ns.

To demonstrate the possible influence of the laser pulse duration on the formation of core-shell nanoparticles, FeAu nanoparticles were generated in acetone with three different laser sources and pulse durations of 120 fs, 10 ps, and 8 ns. HAADF-STEM measurements combined with EDX line scans were conducted for representative particles within the samples. Interestingly, the morphology determined by all three experimental setups revealed the formation of Fe@Au core-shell nanoparticles (Fig. 5). In this context, it should be noted that the pulse duration was not the only parameter altered during the experiments. Indeed, the laser fluence and repetition rate and, hence, nanoparticle productivity, significantly deviated between the various setups. Nonetheless, these findings clearly indicate that the formation of core-shell structures during PLAL of FeAu alloys is a universal phenomenon during PLAL and is not limited to ultrashort (femtosecond) pulses.

A very significant characteristic of the obtained Fe@Au core-shell nanoparticles fabricated in acetone and MMA is the absence of oxygen within the particle core. To probe the extent to which the Au shell may protect the iron-rich core from oxidation, representative samples (laser-synthesized in acetone) were exposed to highly corrosive, concentrated hydrochloric acid to oxidize the iron. Etching the core-shell nanoparticles using hydrochloric acid did not result in any significant changes in the particle’s internal phase structure (Supplementary Fig. S12), as verified by EDX. Therefore, we can conclude that the gold shell is dense enough to protect the reactive iron core against oxidation. This feature is beneficial for applications where high particle stability is of paramount importance, e.g., during biomedical imaging. In this context, the laser-generated Fe@Au core-shell particles are definitely superior to their chemically derived analogs, which exhibit iron core oxidation over time^22^.

**Figure 6:**
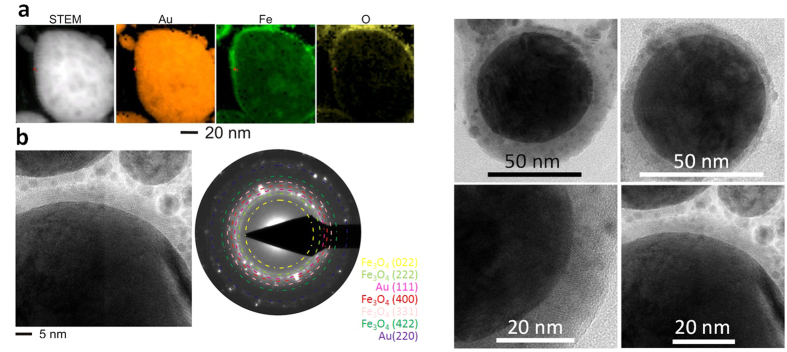
(**a**) STEM-HAADF images (left and right images) and STEM-EDX elemental maps of the nanoparticles formed by the laser ablation of FeAu in water showing the oxidized shell containing iron around the gold core; (**b**) selected-area diffraction pattern confirming the presence of Fe_3_O_4_.

Thus, PLAL of FeAu targets in acetone and MMA yields Fe@Au core-shell nanoparticles; however, when comparable experiments were performed in water, the resulting phase structure was fundamentally different. As shown in Fig. 6, a reverse nanostructure composed of a gold core surrounded by an oxidized iron shell was formed. STEM-HAADF elemental maps (Fig. 6a) show the presence of a gold core and a shell enriched with iron and oxygen. Selected-area diffraction studies and the fast Fourier transformation results supported the existence of the oxidized iron shell (Supplementary Fig. S3), which was primarily composed of Fe_3_O_4_ (Fig. 6b). In addition, no pronounced segregation of gold and iron in the core was noted, indicating that solid-solution nanoparticles may form in the core under these conditions. Electrochemically, the redox potential for elemental iron (−0.447 V)^46^ and the hydrogen overpotential for bulk iron (0.40 V)^47^ indicate that water, in the absence of dissolved O_2_, is capable of (slowly) oxidizing iron; however, it is unlikely that pure water at a neutral pH could be responsible for the rapid and extensive particle oxidation observed in these experiments. Consequently, it is highly likely that dissolved molecular oxygen further increases the oxidative potential of the liquid. To that end, outgassing can be considered as a suitable method for reducing particle oxidation. However, as the Amendola group recently observed during PLAL of an Fe_27_Au_73_ alloy in water, the majority of Fe oxidized during laser synthesis, independent of previous degassing procedures with N_2_, Ar, or CO_2_^37^. Because oxygen removal by inert-gas treatment of water is never complete (typically 0.5–2 mg/L residual oxygen) and because the nanoparticle mass concentration is practically limited by the colloidal stability threshold (typically less than 0.5 mg/mL for ligand-free PLAL-derived gold in water), an iron mass fraction that can be oxidized by residual oxygen after degassing will remain. In addition, it should be noted that the water, acetone, and MMA were not degassed and that the solubility of oxygen in acetone is seven times higher than that in water (solubility: oxygen in water: 5.99 cm^3^/L; oxygen in acetone: 45 cm^3^/L)^48^. Thus, molecular oxygen was present in all liquids during the PLAL experiments. Hence, if dissolved oxygen was the main effector driving the particle internal phase structure, it should be similar in all samples. Consequently, it is likely that the completely different outcomes of intra-particle phase segregation using water or organic solvents are not determined by dissolved oxygen but are more likely to be the result of the intrinsic properties of the respective liquids.

To understand the possible reasons for the solvent-dependent deviations of particle properties, a mechanistic perspective regarding the particle formation process during PLAL is beneficial. During PLAL, the alloy target containing Fe and Au is ablated and the elements are trapped inside a laser-induced plasma plume, which is followed by the formation of a cavitation bubble^49^,^50^. During the PLAL process, plasma formation occurs on a hundreds of nanoseconds timescale after the pulse interacts with the target surface, whereas the lifetime of the cavitation bubble is in the order of 100–200 μs depending on pulse energy^51^,^52^. Recent experiments have shown that the geometry of the bubble is dominated by the plasma plume^53^. Because the plasma plume is hot (up to 7000 K) and subject to strong spatial confinement by the liquid, the Au and Fe atoms released from the target are homogeneously mixed, and segregation is minimized. Consequently, the initial atomic composition inside the plasma and the initial bubble is solely dominated by the target composition. This is supported by the fact that the heats of evaporation for Au (356 kJ mol^−1^) and Fe (355 kJ mol^−1^) are very similar; thus, preservation of the stoichiometry can be assumed^27^. In this context, it should be noted that the PLAL process significantly differs from pulsed laser deposition, where the composition depends on the angle between the laser beam and the target^51^. In a consecutive process, the cavitation bubble expands, whereas the pressure and temperature drastically decrease^52^. This process occurs on a microsecond timescale and induces the formation of initial nanoparticles by crystallization and nucleation^54^. It should be noted that this fast nucleation process is purely controlled by kinetics and that the resulting particles possess a stoichiometry that, next to temperature and pressure, is controlled by the atom concentrations inside the cavitation bubble and, hence, the target composition. Previous experiments have shown that medium components from the solvent, e.g., ions, can be found in the plasma plume^55^. Consequently, solvent effects could occur during the early stages of the particle-formation process and may contribute to a potential ion-induced size-quenching effect^38^,^39^. However, an effect of solvents on particle stoichiometry has not been reported in the literature and was not observed in our study as the EDX analysis of the FeAu nanoparticles and the targets (Supplementary Table S1 and Fig. S9) revealed that similar nanoparticle compositions (i.e., Fe:Au ratios) existed in all solvents. This preservation of the target composition has been previously observed in several nanoparticle systems e.g., PtIr^31^ or AgAu^32^,^34^.

**Figure 7:**
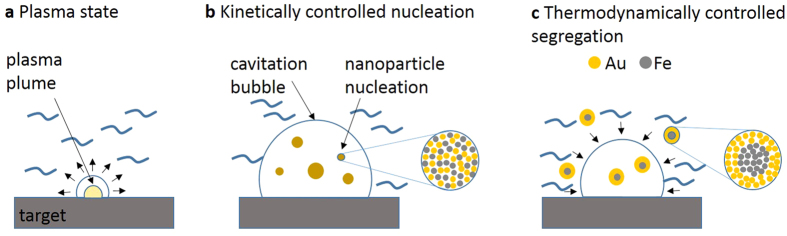
Steps of the nanoparticle-formation mechanism after laser pulse absorption. (**a**) Plasma formation and initial cavitation bubble formation, (**b**) expansion of the cavitation bubble into the liquid and rapid cooling of the ablated matter, and (**c**) cavitation bubble collapse and nanoparticle release into the liquid.

Although the stoichiometry of the target material is generally preserved during PLAL of alloys and is predefined during the initial stages of the particle formation process (kinetically controlled), the internal phase structure of the nanoparticles has been shown to differ depending on the solvent. The De Giacomo group concluded in their review that the dynamics of cavitation bubble shrinking and the transfer of the nanoparticles into solution after bubble collapse allow nanoparticles to be formed in quasi-constant thermodynamic conditions^52^. Consequently, phase segregation and the formation of core-shell structures most likely occur in the solution under thermodynamic control on a timescale far longer than the cavitation bubble lifetime. An illustration of the proposed particle-formation mechanism can be found in Fig. 7.

To further understand the thermodynamically controlled formation of different internal phase structures in different solvents, we compared our findings with data recently reported by Malviya and Chattopadhyay, who examined the synthesis of Cu-Ag alloy nanoparticles by PLAL in an aqueous, polymer-doped medium^56^. They reported Cu-concentration-dependent morphological transitions in the nanoparticles from a defined two-phase nanostructure to a structure with random segregation and, finally, to a core–shell structure. Their hypothesized formation mechanism was rationalized through the thermodynamic modelling of the free energy of phase mixing and the wettability of the alloy phases. Similar to the Cu-Ag system, the iron-gold system shows a quite complex phase diagram, including several phases and miscibility gaps^57^. In the FeAu bulk phase diagram, at room temperature and a composition of approximately 50:50, which corresponds to the target and particle compositions used in this study, the most thermodynamically stable state is complete phase separation, which can be realized via a core-shell structure. Consequently, STEM-HAADF elemental maps of the nanoparticles that were laser-generated in acetone (Fig. 3a), together with the EDX line scans (Fig. 3b) and the electron microscopy images acquired after FIB cutting (Fig. 4c) the particles, confirm the formation of this structure. This finding is in good agreement with the density functional theory (DFT) calculations by Wang and Johnson, who predicted exactly this core-shell preference for FeAu binary nanoparticle systems^58^.

Although this hypothesis conclusively explains why the phase separation and formation of core-shell structures occur, it cannot explain why gold is predominantly formed on the outside of the particle in organic solvents, whereas the reverse composition is generated in water. Here, the interaction of the nanoparticle surface and the solvent, namely, the nanoparticle surface chemistry, may be an important driving force. In organic solvents, gold surfaces are probably formed on the outside of the particle because their interactions with the solvent are favored. Previous experiments reported in the literature indicated that noble metal colloids obtained by PLAL in acetone or ethanol were stabilized by adsorbed enolates or alcoholates, respectively, whereas non-polar solvents could not provide any stabilization^40^. The presence of enolates on PLAL-generated gold nanoparticles in acetone was previously demonstrated by SERS^41^. Both organic solvents (i.e., acetone and MMA) used in the present study contain a keto group. Based on these previous findings from the literature, they may undergo self-polymerization on the gold surfaces of the Fe@Au particles, contributing to the stabilization of these particles in the respective solvents. This self-polymerization process of solvents is more strongly pronounced for αβ-unsaturated carbonyl compounds, such as MMA, compared with acetone. This could explain why particle stabilization and size quenching are more efficient in MMA than in acetone and result in reduced particle diameters in the presence of MMA (Fig. 1d). However, a reverse morphology, with iron oxide on the outside, may be thermodynamically less favored in MMA and acetone, possibly because the potential stabilization mechanisms for hydrophobic gold, e.g., solvent polymerization, may be thermodynamically less favorable for iron oxide. However, the exact nature of the solvent-metal interactions cannot be elucidated based on the current experimental data.

In contrast, in an aqueous solution, a completely different internal phase structure with an iron oxide shell and a gold core forms. As iron oxide is more hydrophilic than elemental gold, its formation on the particle surface in the presence of water is favored. It is well known that metal nanoparticles in aqueous environments form a pH-dependent equilibrium between M-O^−^/M-OH/M-OH_2_^+^ (M = metal), which is the main effector dominating their surface chemistry^59^,^60^.

Based on this theory, we can assume that the chemical interactions between nanoparticles and solvents are an important driving force governing the particle morphology of FeAu in particular - Fe50Au50 bimetallic nanoparticles. This leads to significantly different surface chemistries with either an iron oxide shell or a gold shell, which is probably responsible for the deviating surface charge densities and, hence, the different zeta potentials observed in the different solvents (Fig. 1d). A similar mechanism was previously reported by Mayrhofer *et al.,* who observed a surface segregation of intermetallic PtCo nanoparticles that formed core-shell structures in the presence of carbon monoxide^61^. Because the adsorption enthalpy of carbon monoxide with Pt is more negative than that with Co, Pt segregates on the surface of the nanoparticles, resulting in core-shell structures with a Co core and a Pt shell. Further examples of the surface segregation of bimetallic nanoparticles resulting from chemical reactions at the surface can be found in the literature^62^,^63^,^64^.

**Figure 8:**
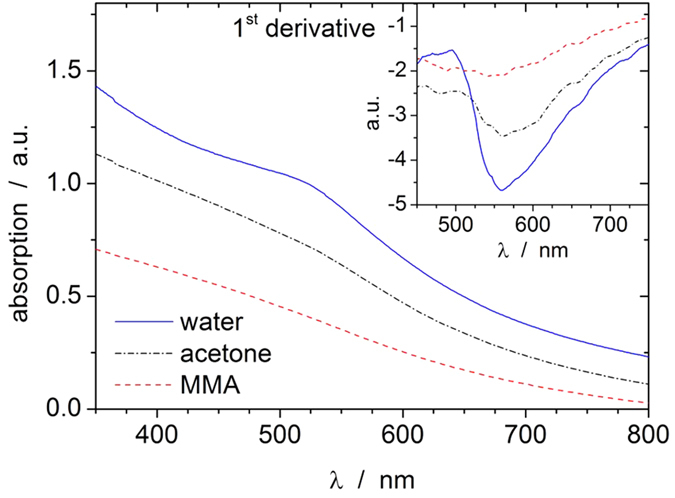
UV/Vis absorption spectra of laser-generated iron-gold nanoparticles in water, acetone, and MMA.

Subsequently, the optical and magnetic properties of these novel hybrid FeAu nanoparticles were evaluated. Representative UV-Vis spectra are shown in Fig. 8 and depict a broad extinction in the visible spectral range superimposed by a gold plasmon resonance peak. The peak position can be visualized more clearly by taking the first derivative, as shown in the inset image in Fig. 8. For all used solvents, the plasmon resonance band is located at approximately 530 nm. Although these surface plasmon resonance (SPR) peaks are relatively weak compared to those of pure gold nanoparticles, the signals in water are significantly larger than those in acetone and MMA. These findings are in accordance with those of previous experiments by Zhang *et al*.^44^. This study also reported a dampening of the SPR maximum in the case where Au was alloyed with Fe in a core-shell structure, and these experimental data were in good agreement with simulations. It should be noted that the observed spectra for the Fe@Au system significantly deviate from those of Fe_3_O_4_@Au^65^, Co@Au^66^, and FePt@Au^67^, where more intense, red-shifted SPR peak signals were observed. However, compared with different materials, both the particle sizes and shell thicknesses found in those studies were significantly different from those found here, which makes comparisons rather difficult.

**Figure 9:**
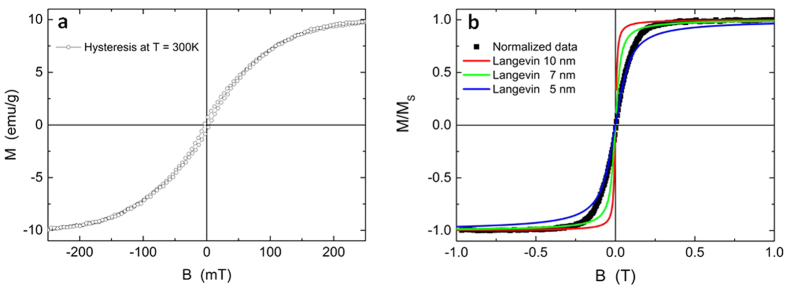
(**a**) Magnetic hysteresis loop of laser-generated FeAu nanoparticles in acetone at T = 300 K. (**b**) Normalized experimental data from (**a**) and Langevin simulations for different Fe core diameters at T = 300 K. Note that different field ranges are displayed.

In addition to the optical properties of the Fe@Au nanoparticles, the magnetic properties of the bimetallic nanoparticles were also characterized. The magnetic measurements of nanoparticles formed by laser ablation in acetone are depicted in Fig. 9. A small open hysteresis with a coercive field of 4 mT was observed (Fig. 9a). The saturation magnetization of 10.3 emu/g was reached at field strengths exceeding 500 mT (Fig. 9b). Considering the size distribution of the Fe cores, with diameters of 5–75 nm (Fig. 1, Supplementary Fig. S1), different magnetic responses from (i) superparamagnetic, (ii) single-domain blocked, and (iii) multidomain nanoparticles would be expected at T = 300 K. Assuming simple estimates using bulk values, we expect superparamagnetic nanoparticles below Fe core diameters of 16 nm and multidomain particles above 50 nm^68^ Thereby, the coercive field is maximized for diameters near the multidomain limit. However, the overall shape of the hysteresis loop in Fig. 9a with a small coercive field, an almost negligible remnant magnetization, and large saturation fields suggests that most of the magnetic response originates from superparamagnetic particles. Even though it is well known that particles with Fe at% <25% exhibit paramagnetic properties, this consideration is not relevant in this system as the particles all had iron enriched cores (compare Fig. 4). Figure 9b presents the normalized experimental data together with simulated Langevin functions for 5 nm, 7 nm, and 10 nm Fe core diameters. An unambiguous demonstration of the origin of the magnetic features would require detailed temperature-dependent measurements, which are beyond the scope of the present contribution. Although none of the simulations accurately fit the experimental data, it is clear that the dominating magnetic signal arises from small nanoparticles with iron core diameters well below 10 nm. This discrepancy compared to the Fe core size distribution measured using TEM (15 nm, Fig. 4d) could be explained by either significantly reduced magnetic anisotropy in the FeAu nanoparticles and a reduced magnetization of the Fe core (by forming Fe-rich alloys) or by small Fe particles surrounded by Au inside the particle core.

## Conclusions

In conclusion, the presented work describes a suitable approach for the one-step synthesis of core-shell Fe-Au nanoparticles by pulsed laser ablation in liquids without the use of reducing agents or organic stabilizers. A detailed elemental analysis revealed that the particle internal phase composition can be controlled by the chemical interactions of the particle surface with its environment. This well-established correlation is exploited to control the nanoscopic particle structure by varying the applied solvents. In the case of the FeAu system, organic solvents able to stabilize gold surfaces are used to produce a gold shell with an iron core. In contrast, aqueous media favor the formation of a hydrophilic oxide shell on the particle surface. Solvent-controlled core-shell nanoparticles are the predominant species formed in both cases. These findings could offer fundamental insights into the particle-formation process during PLAL, indicating that solvent-metal/metal oxide interactions are a key factor dominating the internal phase composition of alloy nanoparticles because surface-metal interactions drive elemental segregation. In addition to fundamental insights into the particle formation process, this work also highlights the synthesis of a hybrid material composed of an Fe@Au core-shell structure in acetone. These particles exhibit outstanding oxidation resistance and moderate magnetic (saturation magnetization 10.3 emu/g) and plasmonic properties. Although these features may seem inferior relative to, e.g., the magnetic properties of pure Fe or Fe_3_O_4_ nanoparticles (saturation magnetization: 218 emu/g)^69^ or the plasmonic properties of pure Au nanoparticles, the combination of these features into one entity could open up novel avenues for applications such as biomedical dual imaging. In addition, the excellent oxidation resistance of the material could be beneficial during long-term applications, where high signal stability is paramount. Moreover, the dense gold shell covering the iron core can be easily conjugated by functional biomolecules via thiol linkers^11^,^13^. Other promising applications of Fe@Au nanoparticles include conductive, transparent coatings, for which recent experimental studies have found them to have outstanding electrical conductivity^70^.

## Additional Information

**How to cite this article**: Wagener, P. *et al*. Solvent-surface interactions control the phase structure in laser-generated iron-gold core-shell nanoparticles. *Sci. Rep.* **6**, 23352; doi: 10.1038/srep23352 (2016).

## Supplementary Material

Supplementary Information
