# Supplementary material for: Solvent-surface interactions control the phase structure in laser-generated iron-gold core-shell nanoparticles
Source: Sci Rep. 2016 Mar 23;6:23352. doi: 10.1038/srep23352 (PMC4804215; doi:10.1038/srep23352)
Supplement: Supplementary Information [file srep23352-s1.doc]

Supplementary Information

**Solvent-surface interactions control the phase structure in laser-generated iron-gold core-shell nanoparticles**

Philipp Wagener, Jurij Jakobi, Christoph Rehbock, Venkata Sai Kiran Chakravadhanula, Claas Thede, Ulf Wiedwald, Mathias Bartsch, Lorenz Kienle, and Stephan Barcikowski*

**Table of content**

1. Particle size, shell thickness, and composition distribution of FeAu nanoparticles laser-generated in acetone
2. Electron microscopy of FeAu nanoparticles laser-generated in MMA
3. Electron microscopy of FeAu nanoparticles laser-generated in water
4. Particle size distributions of FeAu nanoparticles derived from TEM images for all solvents
5. Structure and morphology of FeAu nanoparticles in acetone
   1. EDX-line scans of solid particles
   2. FIB cut preparation
6. Characterization of the target material
   1. Elemental composition
   2. Magnetic properties
7. Etching of FeAu core-shell nanoparticles
8. Stability of laser-generated Fe-Au nanoparticle in acetone

*Contact author: stephan.barcikowski@uni-due.de

**1. Particle size and shell thickness distribution and composition of FeAu nanoparticles laser-generated in acetone**

STEM-HAADF (Scanning transmission electron microscopy – high angle annular dark field) images of FeAu nanoparticles, laser-generated in acetone. They show the core-shell structures for the majority of all detected nanoparticles (>90%). Intermetal nanoparticles are present but less frequent. As shown in Fig. S1, core diameter and shell thickness of each nanoparticle within the STEM image have been measured. The resulting histograms are plotted in Fig S1 and reveal an average shell thickness of about 3 nm and an average core diameter of about 15 nm.

**Fig. S1**: Laser-generated FeAu nanoparticles in acetone: Histograms of shell thickness (left) and core diameters (right).

STEM - EDX analysis on five different nanoparticles show the following results:

Table S1:

|  | Fe(K) | Au(L) |
| --- | --- | --- |
| Np1 | 46.2 | 53.8 |
| Np2 | 45.9 | 54.1 |
| Np3 | 46.9 | 53.1 |
| Np4 | 47.1 | 52.9 |
| Np5 | 46.9 | 53.1 |
| **Average** | **46.6 +/- 0.5** | **53.4 +/- 0.5** |

Thus, the target material composition (Fe_44_Au_56_, see section 4) is nearly preserved during laser ablation achieving nanoparticles with Fe_47_Au_53_.

**2. Electron microscopy of FeAu nanoparticles laser-generated in methyl methacrylate (MMA)**

TEM and STEM images taken of FeAu nanoparticles that were generated by laser ablation in MMA did not differ significantly from those generated in acetone. The chemical analysis in STEM using EDX and HAADF detectors confirmed the core-shell structure (core-Fe and shell-Au) without any trace of oxidation.

**Fig. S2** STEM-HAADF-EDX of laser-generated Fe@Au core-shell nanoparticles in MMA.

**3. Electron microscopy of FeAu nanoparticles laser-generated in water**

In contrast to acetone and MMA, pulsed laser ablation in water yielded core-shell nanoparticles with reverse microstructure: gold core and iron oxide shell. The visibility of the shell is enhanced in the electron micrograph when the reflections from the core material are filtered by fast Fourier filtering, thereby suppressing the contrast of the Au core as can be seen in Fig. S3d.

**Fig. S3** HR-TEM of laser-generated Au@Fe_3_O_4_ core-shell nanoparticles in water using different magnifications (**a-c**), image detail using Fast Fourier filtering (**d**) and selected area diffraction (**e-f**).

**5. Structure and morphology of FeAu nanoparticles in acetone**

**a) EDX line scans of solid nanoparticles**

As reported in section 1, most of the observed binary nanoparticles show a core-shell structure whereas intermetallic (solid solution) nanoparticles appear only occasionally. These internal structure and morphology can be differentiated by line scans shown in Figure 2 and 3 in the manuscript.

By calculation the share of the element-specific signal normalized by the total signal, the difference is even clearer (Fig. S4 and S5). The core-shell nanostructures show iron-enrichment in the core and gold-enrichment in the shell. In contrast to this, intermetallic particles show the same ratio over the whole nanoparticle as typical for solid solution (see Ref. 32 in manuscript).

**Core-shell nanoparticle Intermetal nanoparticle**

**Au**

**Fig. S4** Ratio of EDX gold signal normalized to total signal

**Fe**

**Fig. S5** Ratio of EDX iron signal normalized to total signal for laser-generated nanoparticle in acetone.

The core-shell nanoparticle was further characterized by integration of the EDX-signal of specified regions. Fig. S6 depicts a HRTEM image of a core-shell nanoparticle fabricated in acetone with specified regions in core and shell used for EDX analysis. All regions in the shell show a gold signal without any contributions by iron. Inside the core iron and gold are detected.


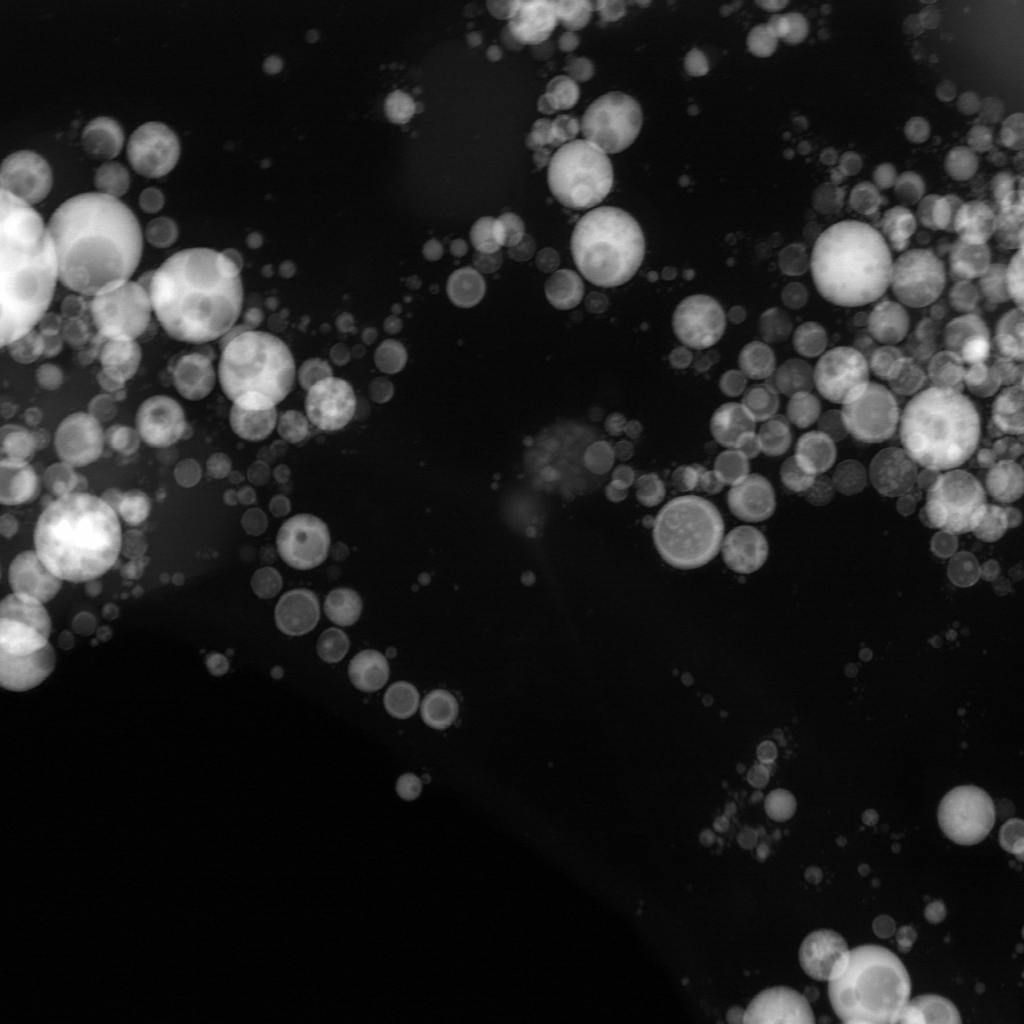


2

1

4

3

2

3

1

**Fig. S6**

HRTEM of an Fe@Au core-shell nanoparticle laser-generated in acetone and regions (shell: 1-3, core: 4) for EDX analysis (Box sizes are 7x7 nm)

EDX spectra of marked sites are shown on same page / below

Shell

Shell

Shell

Core

**b) FIB cut preparation**

An FEI Helios Nanolab combined Focused Ion Beam (FIB)/SEM instrument was used for thin lamella preparation for subsequent TEM-EDX analysis. TEM EDX measurements (Fig. S8) were carried out using a FEI Tecnai F20 transmission electron microscope (acceleration voltage 200 kV).

For preparation of the FIB cutting, the colloidal Fe-Au nanoparticles in acetone are dropped on a silicon wafer and dried on the air for several minutes. To protect the nanoparticles for cutting steps, an appropriate site with sufficient nanoparticle concentration was sputtered with Pt by using the FEI Helios Nanolab FIB instrument (Fig. S7a). Afterwards holes beside the sputtered area are ablated with FIB, leaving a thin lamella with nanoparticles embedded between silicon substrate and Pt-coating (Fig. S7b and S7c). The sliced lamella was incorporated with the tip of Pt wire by sputtering and cut out of silicon wafer (Fig. S7d). In the next step, the lamella was placed on FIB lift-out TEM grid fixed by sputtering with Pt and cut from the wire tip. On the end, the fixed lamella was thinned by the FIB until its tip has reached a size ≥50 nm. Figure S7f shows a side view in STEM mode of lamella. The element distribution of FIB sliced nanoparticles was proved using the TEM EDX line scan and shown in the Fig. S8.

**Fig. S7** Nanoparticle cross-sectioning: SEM micrographs of nanoparticles during FIB cut preparation steps with (**a**) protective Pt coating sputtered on the Fe@Au nanoparticles (dried drop of nanoparticle colloid synthesized in acetone), (**b-d**) FIB cutting of nanoparticle-containing lamella, (**e**) top view of thinned lamella with sliced nanoparticles, (**f**) STEM side view (cross section) of the thinned lamella with particles cut-through.

**Fig. S8** Confirmation of core-shell structure of laser-generated Fe@Au nanoparticle in acetone after particle has been cut: (**a**) STEM-HAADF micrograph, where the interface between the core and the shell is even clearer visible in this FIB-cut cross section than in the uncut HRTEM (Fig S6). (**b**) TEM EDX line scan of FIB-sliced Fe@Au core-shell nanoparticle. Regarding the EDX line-scan please note that the probe beam is retrieving signal of an excited area of a few nanometers, so even a steep element contrast scanning across the phase boundary of the core and the shell would lead to a smoothed signal with elemental contribution from both phases.

**6. Characterization of the target material**

We characterized the target material composition and magnetic properties by EDX-measurements and by a vibrating sample magnetometer, respectively.

- 1. **Elemental composition**

The elemental composition of the target was measured by EDX at three target positions and resolutions.


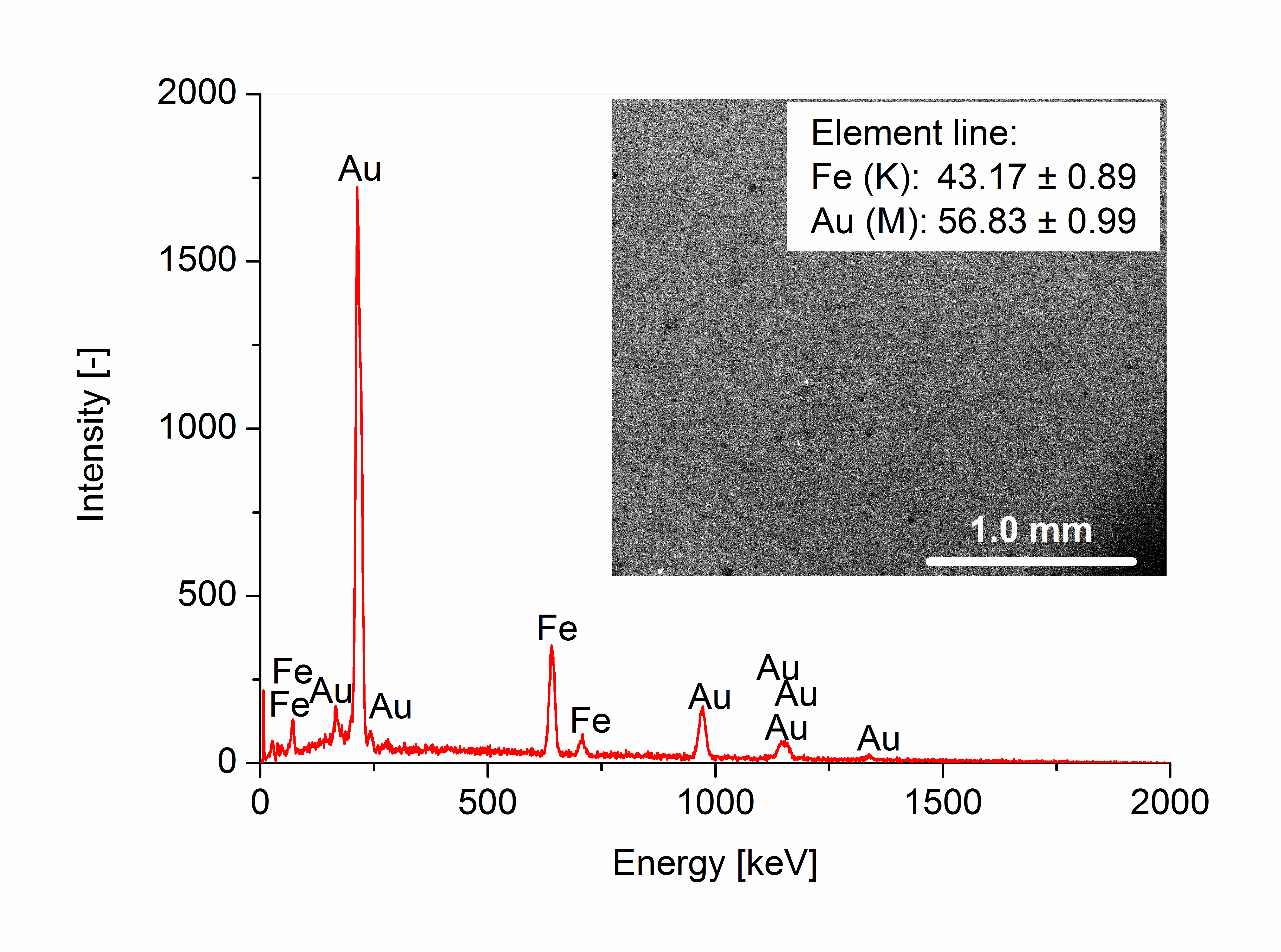


**Fig. S9**

SEM micrographs of used target and detected EDX spectra


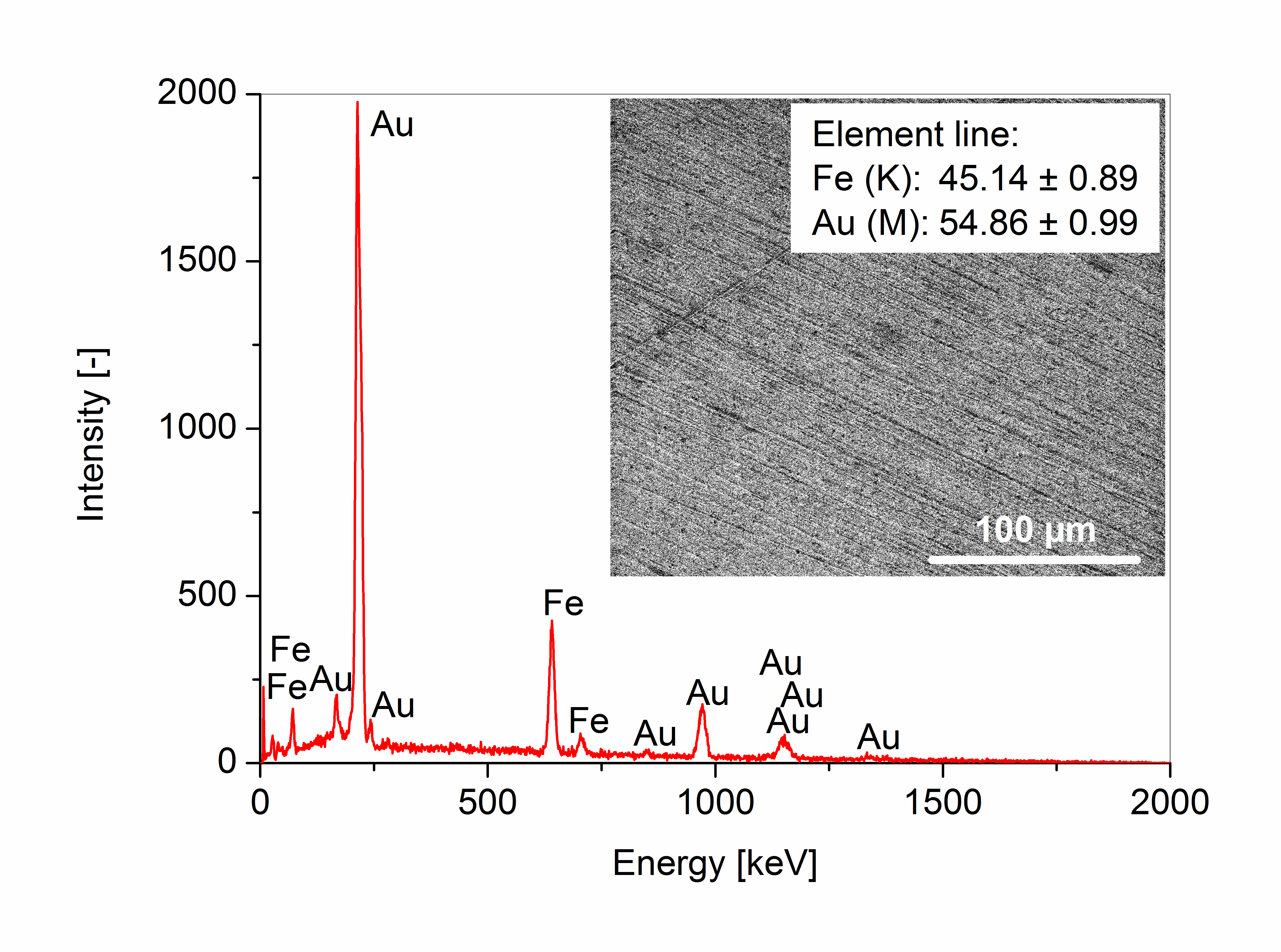

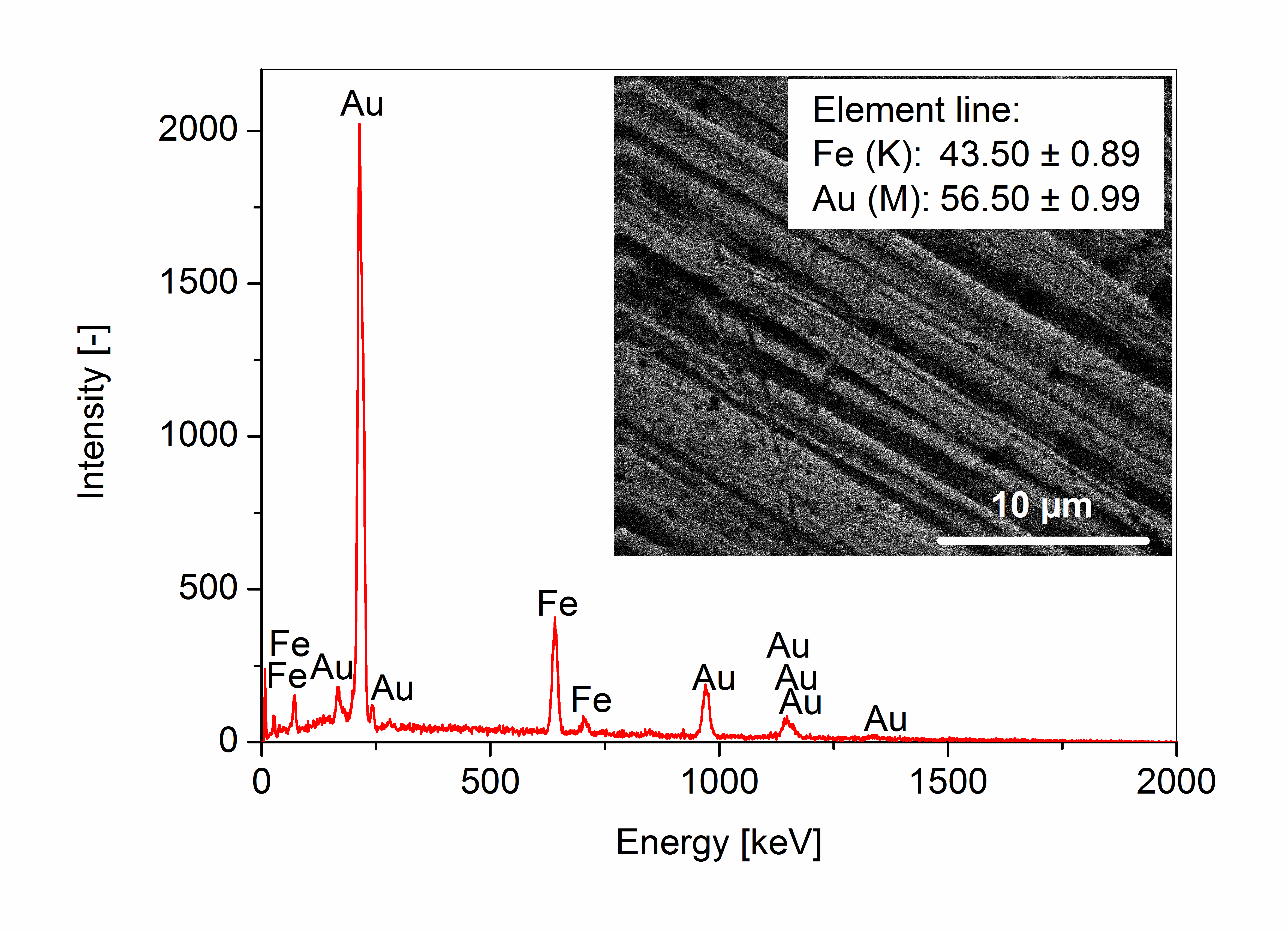


The arithmetic mean of all three measurements results in a target composition of Fe_44_Au_56_.

- 1. **Magnetic properties**

The magnetization of a 3.6 mg fragment from the Fe_44_Au_56_ target was measured by a vibrating sample magneto-meter (Fig. S10). The data has been corrected for demagnetization by *N_d_* = 0.02, corresponding to the ferromagnetic behaviour of the sample and its dimensions (Fig. S11).


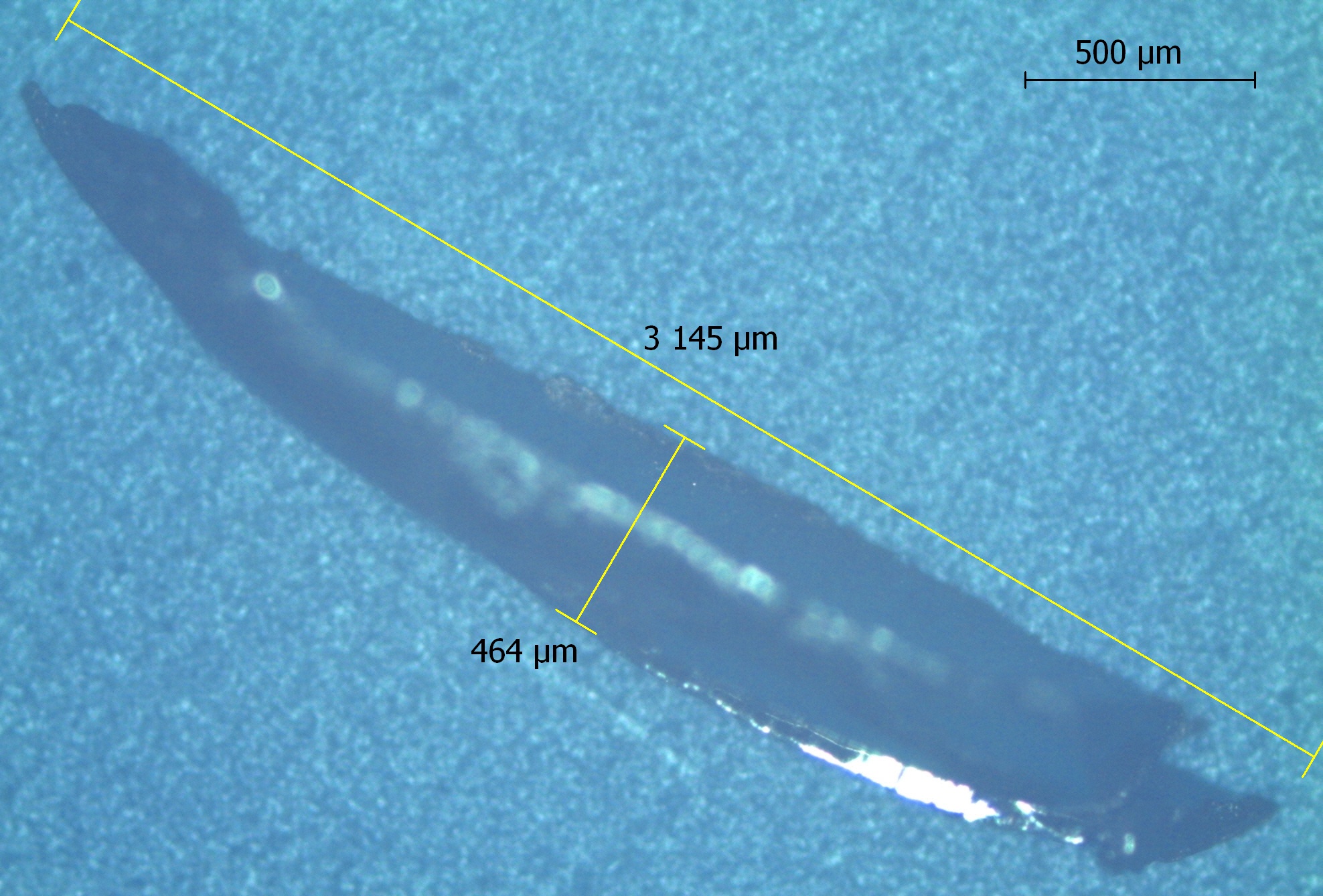


**Fig S10** Micrograph of a fragment from the Fe_44_Au_56_ target.


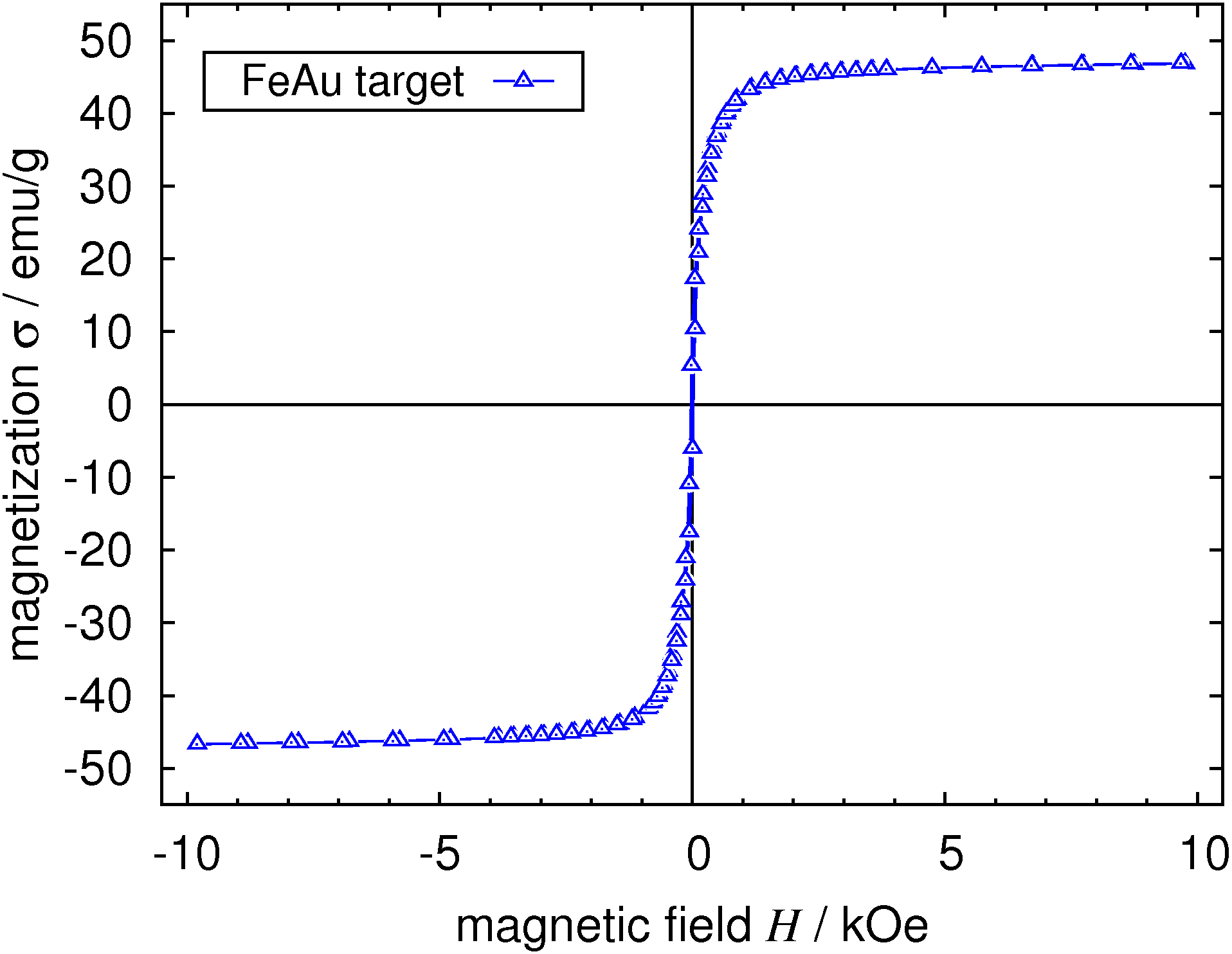


**Fig. S11**

Magnetization of used FeAu target

**7. Etching of FeAu core-shell nanoparticles**

To prove the protecting gold shell around the iron core, we etched iron-gold core-shell nanoparticles with diluted (10 %) and concentrated (37 %) hydrochloric acid. After washing, the etched particles were analysed by electron microscopy for their elemental composition. As can be seen in Fig. S12, the core-shell structure is still visible after etching. Thus, the gold shell might be considered as an effective protection of the iron core against oxidation.

**Fig. S12** TEM micrograph and elemental composition of core-shell Fe@Au nanoparticles after etching with conc. HCl.

The productivity of used 1.5 W femtosecond laser is about 8 mg/h of Fe-Au-Nanoparticles.

**8. Stability of laser-generated Fe-Au nanoparticle in acetone**

**Fig. S13** Long-term stability of laser-generated Fe-Au nanoparticles in acetone measured by time-resolved UV-Vis spectroscopy.
